# Progress in Radiology of the Stomach, Intestine and Appendix in 1913

**Published:** 1914-04

**Authors:** 


Progress in Radiology of the Stomach, Intestine and Appendix in 1913. Condensed Translation from *Le Progres Medical,* December 20, 1913.

Bensaude has advocated the use of barium sulphate, as less expensive and less toxic than the subnitrate of bismuth. Any such substance may, however, prove dangerous by its weight. Thus, 60-100 grams of bismuth carbonate may cause ptosis of the stomach. Perforation of a cancerous ulcer of the stomach and volvulus of the small intestine has been noted from an emulsion of barium sulphate.

Gregory Cole of New York and Howard Pirle representing Rosenthal of Munich, showed cinematographs of the stomach by the bismuth-Roentgen method. Gösta Forsell's Atlas of Normal and Pathologic Anatomy studied by the X-ray, deserves mention.

Bonniot and Rideaux advocate filling oesophageal diverticula with bismuth, in emulsion or cachet, and then watching the stream of bismuth swallowed through the ordinary course of the oesophagus, as more reliable means of diagnosis than the two sound method or the simple administration of cachets of bismuth.

G. Lion has checked the X-ray method very simply, by administering 20 grams of bismuth subnitrate to a dog and killing it after 40 minutes, and making direct inspection. The entire surface of the stomach is found uniformly powdered and the bismuth is well mixed with the meal, though not so uniformly as it is spread over the surface. Sections of mucous membrane were also examined microscopically, after fixation with alcohol, showing the subnitrate and some characteristic crystals of oxychlorid of bismuth.

Paul Carnot, referring to the warning of Beclere and Meriel that blanks in the radiologic image of the stomach cannot be considered pathologic, as one cannot be certain that the walls of the stomach, originally in contact, have been uniformly separated and coated with bismuth, advocates massage to insure that the bismuth comes into contact with all parts of the stomach. (Note: We have personally encountered misinterpretation of radiographs to indicate hour-glass contraction when the absence of bismuth was due simply to jumping from one part of the stomach to another, on account of rapid peristalsis· In one of these cases, the hour glass contraction seemed beyond question, when any one radiograph was inspected *but* the contraction was at a different zone in different pictures taken within a few minutes of each other. Fluoroscopic examination clearly excluded hour glass contraction and showed the rapidity and depth of the contractions to an almost incredible degree, while the general contour of the stomach, with allowance for the passing waves, corresponded exactly to the auscultatory percussion area, just as was found in original experiments in 1897.)

Walther mentions radiographs apparently indicating gastric diverticula, which were excluded by section and inspection. Various communications regarding preferable posture, the gas ball of the stomach and the location of a hair ball by X-ray, combined with manipulations, already abstracted, are mentioned.

Leven and Barret have shown that milk, as sucked by the infant passes the pylorus almost immediately, so that the amount and intervals of nursing depend on the capacity of the small intestine. Siegert states that the intestinal digestion of milk in the nursing, occupies about 3½ to 4 hours and that this is the proper interval between nursings.

The question as to whether bismuth adheres especially to an ulcer is still in dispute, some authorities giving elaborate rules as to the shadows observed, others denying that ulcer can be diagnosed regularly by X-ray methods, although admitting that an old, cicatrized ulcer, with cribriform scar, tends to retain bismuth.

Piret mentions an ingenious device for confirming the diagnosis of appendicitis. A metal bar is strapped over the painful spot. Radiography, several hours after the ingestion of bismuth, shows the ileo-caecal angle and if the two shadows coincide, the appendix is pretty definitely incriminated. Fallacies may, however, occur.

Friedel and Jaugeas emphasize the fact that, aside from operative inspection, the sigmoid is accessible only to sigmoidoscopy and radiologic investigation, the former applying only to the first part of the sigmoid (recto-sigmoid) while the latter with the oblique screen or plate, allow one to note the form, tonicity, length, bends and adhesions of the upper sigmoid. (Note: The more one works with the sigmoidoscope and the more one notes the appearance of this part of the bowel at necropsies and operations, the less significant does the term *sigmoid* become and the less importance one is disposed to attach to ordinary anatomic descriptions. We do not remember ever to have seen a sigmoid which resembled strongly either the Greek letter sigma, or the S Romanum, as the Germans call this part of the intestine. As to sigmoidoscopy, one cannot estimate exactly how much the bowel has invaginated itself over the instrument and one can only say that, at such a distance above the anus, such and such lesions have been encountered.)

